# Associations among Solo Dining, Self-Determined Solitude, and Depression in South Korean University Students: A Cross-Sectional Study

**DOI:** 10.3390/ijerph18147392

**Published:** 2021-07-10

**Authors:** Sunjoo Jang, Haeyoung Lee, Seunghye Choi

**Affiliations:** 1 Red Cross College of Nursing, Chung-Ang University, Seoul 06974, Korea; icedcoffee@cau.ac.kr; 2 College of Nursing, Gachon University, Incheon 21936, Korea; hera1004@gachon.ac.kr

**Keywords:** solo dining, self-determination, depression, dietary habits, eating behavior

## Abstract

Although solo dining motivated by self-determined solitude can be a positive and healthy experience for individuals, solo dining that is not motivated by self-determined solitude can trigger physical and mental health problems. This study examined the associations among solo dining, self-determined solitude, and depression in university students. Accordingly, an online survey was conducted on 372 university students. The results show that students who live alone, those in poor health, and those with more frequent solo dining experiences had higher depression scores than others. Whereas satisfaction with solo dining was high when voluntary solitude was high, female students displayed higher depression scores when they had low self-determined solitude or high non-self-determined solitude, and when they had a higher frequency of eating lunch alone, compared to their male counterparts. University undergraduates who live and dine alone, owing to non-self-determined solitude, are highly vulnerable to mental health problems, including depression. Hence, interventions that foster social connectedness and entail the identification of factors accounting for students’ non-self-determined solitude should be developed.

## 1. Introduction

Eating a meal is a social activity that strengthens individuals’ relationships with others and has a significance beyond consuming food [1]. However, the rapid growth of single-person households and the sociocultural emphasis placed on an individualized lifestyle may have caused an increase in the number of people who enjoy solo dining [2]. In 2018, single-person households accounted for 29.3% of all households in South Korea [3], and an upsurge in the number of such households has caused a spike in the rate of solo dining (i.e., people eating alone) [3].

Eating alone is influenced by not only individual preferences but also social and cultural factors [4]. South Korea’s prevalent cultural theme is characterized by a vertical and collectivistic configuration [5]. Hence, in the past, eating alone was taboo; however, recent cultural changes have increased the acceptability of eating alone. The reasons for solo dining vary across age groups. Currently, people in their 20s and 30s hold more positive attitudes about eating alone compared to those in their 40s and 50s in South Korea [6]. A survey conducted on workers in their 20s through 60s revealed that the main reason for eating alone was “to eat at my own pace” (24.2%) among people in their 20s, and voluntary solo dining was more evident among people in this age group; other age groups mentioned a “lack of time” or “having no one to eat with” as the main reasons for eating alone [6].

People who eat alone can be classified into willing and unwilling solo groups depending on their self-motivation [7]. Solitude refers to a lack of social interactions; earlier psychological studies mostly focused on the negative aspects of solitude. However, the phenomenon of being alone is complex [8]. One notable predictor of positive solitude experiences is self-determined motivation. Burger [9] reported that people who prefer solitude spend more time alone, perceive the experience to be a more positive one, and feel less bored and lonely when alone compared to those who do not prefer solitude.

According to self-determination theory, autonomous regulation develops from not only external pressures, prompts, and temptations but also a person’s emotions, impulses, and urges [10]. An earlier study ascertained that only non-self-determined solitude (NSDS) positively correlated with loneliness and social anxiety, whereas self-determined solitude (SDS) demonstrated no relationship with these negative outcome variables [8]. According to this perspective, voluntary solo dining (i.e., solo dining from SDS) is not depressing or lonely but rather a fun experience, and solitude can be constructively used to develop cognitive and emotional skills [11]. Furthermore, a person’s capacity for solitude may become evident in adolescence and blossom at the beginning of adulthood [8]. Therefore, it is crucial to promote a healthy manifestation of SDS during this period.

In a study on college students, eating alone had a significant positive correlation with depression [12]. However, most studies that investigated the relationships between eating alone and the occurrence of mental health problems, particularly depression, focused on the elderly [13,14]. In particular, it is difficult to find a study that analyzes the association among solo dining, motivation for solitude, and depression in university students.

University students can perform solo dining voluntarily or involuntarily. To investigate the relationship between solo dining and depression among South Korean university students, it is necessary to examine students’ solo dining experience, including solitude. Thus, we investigated the current trend of solo dining among university students and the factors associated with their solo dining tendency, including the motivation to seek solitude, and depression. In this manner, we aim to provide foundational data to develop education and intervention programs that promote healthy dietary behaviors among students.

## 2. Materials and Methods

### 2.1. Study Design

This study employed a cross-sectional survey design to assess the associations among solo dining, solitude (self-determined/non-self-determined), and depression in South Korean university students.

### 2.2. Setting and Participants

This study enrolled university students with solo dining experience (i.e., for all instances of eating alone). Since this study considered mental health parameters, the participants who had been diagnosed with a mental health disorder, such as depression or anxiety, and were currently being treated were screened out. The survey was conducted in November 2020, a time when the number of newly confirmed coronavirus disease 2019 (COVID-19) cases per day temporarily fell below 200 before increasing exponentially in South Korea. The sample size was determined using the G*Power program developed by Faul, Erdfelder, Buchner, and Lang [15], and power analysis was performed for multiple regression analyses, with a medium effect size of 0.15, a significance level of 0.01, a power of 0.95, and 15 predictors (i.e., 7 general characteristics, 6 solo dining-related characteristics, SDS, and NSDS). The minimum sample size was computed as 252, and an online survey was administered under the assumption of a maximum withdrawal rate of 30%. The data of 372 students were included in the final analysis.

### 2.3. Measurements

The relevant variables were measured using specific instruments.

#### 2.3.1. General Characteristics

The general characteristics of participants included gender, school year, age, living arrangement, socioeconomic status (SES), and subjective health status.

#### 2.3.2. SDS/NSDS

An adapted and validated version of the Motivation for Solitude Scale [16], which was developed by Nicol [17], was used. This tool consists of two subscales: SDS and NSDS subscales. The SDS subscale consists of 29 items, each rated on a four-point Likert scale, with higher scores indicating greater self-determined motivation. The NSDS subscale consists of 25 items, each rated on a four-point Likert scale, with higher scores indicating greater non-self-determined motivation. In this study, the Cronbach’s alpha reliability coefficients for the SDS and NSDS scales were 0.931 and 0.952, respectively.

#### 2.3.3. Depression

Depression was measured using the Center for Epidemiological Studies-Depression Scale (CES-D). The CES-D is a self-report tool developed by Radloff [18] for an epidemiological study on depression in the general population. The current study used the comprehensive Korean version of the CES-D—a validated tool that integrates the three Korean versions of the CES-D [19]. The tool consists of 20 items rated on a four-point scale (0–3) to assess the symptoms experienced by individuals in the preceding week. Total scores range from 0 to 60, with higher scores indicating greater levels of depression. The tool measures four factors: depressive affect, positive affect, somatic symptoms, and interpersonal relationships. In this study, the tool’s Cronbach’s alpha reliability coefficient was 0.918.

#### 2.3.4. Solo Dining-Related Characteristics

The frequency of solo dining was defined as the number of breakfasts, lunches, and dinners eaten alone by participants in the previous week. Some examples of the reasons for solo dining were to save time, having no one to eat with, to save money, and for comfort. Moreover, the place, duration, and perception of and satisfaction with solo dining were measured using a visual analog scale (0–10).

### 2.4. Data Collection

We used the services of an online survey company for the countrywide recruitment of students. The company used a preexisting online panel sample, where the students in the panel lists were originally recruited to the panel using random sampling methods. Data were collected from the students who voluntarily participated in the survey conducted in November 2020. We did not need to obtain written consent because the study did not consider sensitive factors that might have provoked participants to decline participation, and their participation would not have posed any risk greater than in everyday life. Nonetheless, before starting the online survey, participants were asked to specify in a checkbox whether they consented to participate.

### 2.5. Data Analyses

Statistical analyses were conducted using SPSS 24.0 (IBM, Armonk, New York, NY, USA). Participants’ general and solo dining-related characteristics and SDS, NSDS, and depression scores were analyzed using descriptive statistics. The differences in participants’ solo dining frequency, perception, satisfaction, and depression, according to their general characteristics, were analyzed using χ^2^ tests, *t*-tests, and analyses of variance. For post hoc comparisons, Scheffé’s tests were conducted. Further, to determine the associations among SDS, NSDS, and depression, Pearson’s correlation analyses were conducted. Finally, a multiple regression analysis was conducted to identify the impact of general and solo dining-related characteristics, SDS, and NSDS on depression. For the regression model, we developed a causality based on some earlier studies [13,14]. We used the Kolmogorov–Smirnov test to verify the normality of the data, and the Breusch–Pagan test to verify the homoscedasticity of residuals for linear regression.

## 3. Results

### 3.1. Participants’ Demographic and Solo Dining-Related Characteristics

Participants’ general and solo dining–related characteristics are depicted in Table 1. The ratio of male to female students was 1:1. A portion of the participants were fourth-year students (35.8%), and 83.9% were enrolled in school at the time of the study. Most lived with family or relatives (66.9%), and 47.1% perceived themselves to be in good health. Regarding their SES, 43.5% were classified as belonging to the middle class. The weekly solo dining frequency was the highest for lunch (3.70 times/week), and the most common solo dining location was home (68.3%). The prime reason for solo dining, for which the participants could choose up to three answers, was comfort (23.7%).

### 3.2. Comparison of Solo Dining-Related Characteristics and Depression Based on General Characteristics

Table 2 summarizes the association between solo dining-related characteristics and the occurrence of depression according to participants’ general characteristics. There were no significant differences in solo dining frequency and perception regarding satisfaction with solo dining according to gender, school year, school registration status, and SES. The frequency differed according to living arrangements: students living alone showed a significantly higher solo dining frequency (M = 11.19, standard deviation (SD) = 4.12) than those having other types of living arrangements (F = 12.96, *p* < 0.001, df = 3). Furthermore, students with poor health displayed a higher solo dining frequency (M = 10.12, SD = 3.96) compared to those in good health (F = 3.86, *p* = 0.022, df = 2). Male students had lower depression scores (M = 15.76, SD = 10.55) than female students (t = 3.14, *p* = 0.002, df = 1), sophomore students had lower depression scores (M = 14.18, SD = 9.25) than junior or senior students (F = 4.59, *p* = 0.004, df = 3), and students in good health had lower depression scores (M = 14.25, SD = 9.33) than those with poor health or those with moderate health (F = 19.17, *p* < 0.001, df = 2) (Table 2).

### 3.3. Correlations among Variables

Table 3 depicts the correlations among participants’ solo dining-related characteristics, SDS and NSDS scores, and depression. Older students recorded more solo dining experiences (r = 0.278, *p* < 0.001) and ate dinner alone more frequently than their younger counterparts (r = 0.177, *p* = 0.001). Further, solo dining was positively correlated with SDS (r = 0.171, *p* = 0.001), and weekly solo dining frequency was positively correlated with depression (r = 0.139, *p* = 0.007). Students with higher SDS scores were more satisfied with solo dining (r = 0.460, *p* < 0.001) and had lower depression scores (r = −0.134, *p* = 0.009) compared to their counterparts with lower SDS scores.

### 3.4. Factors Influencing Depression

Before performing multiple regression analyses, we evaluated the autocorrelations of dependent variables, and the multicollinearity of independent variables. The Durbin–Watson index indicated independence. The variance inflation factor indicated an absence of multicollinearity (Table 4). Subsequently, in the regression model, we entered the predictor variables related to depression. The regression analysis revealed that gender (β = −0.12, *p* = 0.005), health condition (β = 0.15, *p* = 0.001), eating lunch alone (β = 0.10, *p* = 0.040), satisfaction with solo dining (β = −0.10, *p* = 0.038), SDS (β = −0.15, *p* = 0.002), and NSDS (β = 0.51, *p* < 0.001) significantly predicted depression. In other words, depression was high in female students, students who ate lunch alone, those with low satisfaction with solo dining, those with low SDS scores, and those with high NSDS scores. These predictors accounted for 40.3% of the variance in depression. Further, the Kolmogorov–Smirnov test results (Z = 0.06, *p* = 0.128) satisfy the normality assumption, and the Breusch–Pagan test results satisfy the homogeneity of variance assumption (χ^2^ = 15.09, *p* = 0.088) (Table 4).

## 4. Discussion

This study investigated the current trend of solo dining among university students and the association among solo dining, depression, and motivation for solitude. The most common reason for solo dining was comfort, which indicated the presence of more voluntary solo dining cases than involuntary solo dining cases. The solo dining frequency was the highest for lunch, at 3.7 times a week, and 68.3% of the students said that they eat alone at home. Students chose their home as the most common place for solo dining, although a small percentage selected social distancing due to COVID-19 as the main reason. However, the data collection period indicates that we cannot eliminate the possibility that the solo dining trend was influenced by the restrictions placed in restaurants or the students’ fear of meeting other people in the COVID-19 pandemic situation. In addition, even after excluding the COVID-19 situation, students experienced discomfort caused by others’ perceived negative judgments on eating alone at a restaurant outside one’s house, etc. [20]; moreover, a restaurant’s physical and psychological boundaries affected solo diners’ perceived territoriality [21]. Therefore, the limited number of places outside the house where students can comfortably dine alone could also be a reason why most of them chose the house as a preferred place for solo dining.

Regarding the differences in solo dining-related characteristics according to the participants’ general characteristics, the participants who lived alone or claimed to be in poor health engaged more frequently in solo dining than their counterparts with other living arrangements and good health. In general, people who eat alone have a poor nutritional intake [22] since they commonly consume processed foods or display poor eating habits, such as eating quickly [23,24]. Therefore, stakeholders should pay attention to students who are in poor health and live alone or frequently eat alone. Additionally, researchers should assess the quality of the students’ meals and develop interventions to improve their dietary behaviors.

Regarding correlations among key variables, solo dining frequency was positively correlated with depression. In other words, the higher the frequency of solo dining, the higher the likelihood of participants experiencing depression. Conversely, depressed students are more likely to eat alone than other students. Since the purpose of dining out is both to eat food and promote social fellowship, people generally go to restaurants in couples or groups [25]. Thus, people who eat alone tend to experience high levels of negative emotions such as loneliness and depression, which adversely affect their mental health [22,26,27]. In particular, compared to their Western counterparts, South Korean people are more influenced by such emotions when eating alone, owing to their community-focused culture [28].

However, our results show that participants with high SDS were more satisfied with solo dining and had significantly lower depression scores compared to those with low SDS. People with high SDS chose solo dining voluntarily; thus, they were more likely to be satisfied with solo dining and less likely to experience depression. People who prefer solitude spend more time alone, are more likely to perceive that time positively, and are less likely to feel bored or lonely compared to those who do not prefer solitude [9]. 

In the multiple regression analysis, the effects of solo dining-related factors and solitude on depression, gender (female students), eating lunch alone, SDS, and NSDS were predictors of depression. In other words, depression scores were higher among female students than male students, students with a higher lunch solo dining frequency, and students with lower SDS but higher NSDS scores compared to others. Solitude refers to the absence of social interactions, and earlier psychological studies primarily focused on its negative aspects [29,30]. However, people who have a high preference for solitude are likely to experience solitude positively, and self-determined motivation critically affects such people’s positive solitude experience [8]. In other words, if individuals desire solitude and reap its benefits, they will have a pleasant time being alone. From this perspective, voluntary solo dining—that is, SDS-motivated solo dining—may be a pleasant experience.

Jing et al. [31] found that self-reported depression was severer than usual among university students during the COVID-19 pandemic period. In addition, according to one study [32] that comparatively analyzed the dietary life of South Korean university students before and during the COVID-19 pandemic, students tended to eat alone more frequently and in a pleasanter atmosphere during the COVID-19 pandemic compared to before. Therefore, we should consider the timing of our survey, which was conducted amid the pandemic situation, since the timing could have affected participants’ frequency of eating alone, SDS, and susceptibility to depression.

A recent study that compared the differences in participants’ solo dining attitudes according to their dietary habits and age [3] identified eight factors affecting attitudes regarding solo dining and classified these factors into three categories: “healthy eating alone,” “solo dining,” and “eating alone as a daily routine.” The study observed that people in their 20s and 30s were more likely to fall in the low healthy meal group than in the high healthy meal group. Further, younger adults ate alone as part of a daily routine, and people who had a positive view of solo dining dined out alone more frequently than those with a negative view. In other words, people with positive attitudes toward solo dining and those in the high healthy meal group enjoyed solo dining, whereas those in the low healthy meal group held more negative attitudes toward solo dining [3]. The increase in the depression score with an increase in the lunch solo dining frequency, decrease in SDS scores, and increase in NSDS scores observed in this study can be interpreted in the same light, as well. This means that even young adults can enjoy healthy solo dining if they are motivated by SDS; however, having to eat alone as a daily routine without being motivated by SDS may provoke feelings of depression and lead to unhealthy solo dining.

Unlike in the past, solo dining is now being considered a valuable and meaningful activity that should be performed by individuals to add value to their hectic daily lives [3,27]. Currently, solo diners are fostering a new dining culture by enjoying their time alone. However, it is crucial to note that there are students who are eating alone involuntarily and are at risk of physical and mental health problems; thus, interventions are needed to help them prevent depression and enjoy a healthy diet.

Although there are several advantages to solo dining, an increase in the frequency of eating alone curtails opportunities for individuals to benefit from the support of family and friends [33]. A high frequency of eating with others lowers the risk of health problems and improves health-related quality of life [34]. Therefore, conditions under which college students can enjoy solo dining by voluntary choice should be prepared; further, those students who frequently undergo involuntary solo dining should be supported with interest so that they can enjoy the physical and mental health benefits of meals eaten together with their family and friends. 

This study has a few limitations. First, owing to the cross-sectional nature of this study, we could not infer causal relationships among solo dining, motivation for solitude, and depression. In particular, at the time of conducting the survey (November 2020), South Korea was reporting significant daily increases in the number of newly confirmed COVID-19 cases and the total number of people infected with COVID-19. These numbers might have affected the association among variables. Thus, longitudinal studies are required to substantiate causality. Second, participants were recruited without limiting their eligibility to attendance in a specific university, and the sample was not representative of all university students, which limits the generalizability of the findings. Future studies should recruit a larger and more representative sample for further investigation. Third, although we examined the solo dining frequency and health conditions of students, because the data were collected using a self-report questionnaire, there was a potential for recall bias and social desirability bias. Finally, the study did not investigate the content and quality of solo meals; therefore, subsequent studies should employ objective assessments of solo dining to establish its effects on physical and mental health.

## 5. Conclusions

Those who live alone, those in poor health, and those who frequently eat alone displayed high depression scores. Whereas students with high SDS scores were highly satisfied with solo dining, female students, students with low SDS and high NSDS, and students who frequently ate lunch alone had high depression scores. Voluntary solo dining may be relaxing to many; however, frequent involuntary solo dining may contribute to depression. Hence, interventions should be developed to support at-risk students, such as those that involve identifying the causes of these students’ NSDS and promoting social connectedness.

## Figures and Tables

**Table 1:** Participants’ general and solo dining-related characteristics (*n* = 372).

Variable	Category	*n* (%)	M (SD)
Gender	Male	186 (50)	
	Female	186 (50)	
Age (year)			22.36 (2.20)
Year	1	61 (16.4)	
	2	87 (23.4)	
	3	91 (24.5)	
	4	133 (35.8)	
School registration status	Registered	312 (83.9)	
	On leave	60 (16.1)	
Living arrangement	Live in a dormitory (group life)	37 (9.9)	
	Live alone	72 (19.4)	
	Live with friends	14 (3.8)	
	Live with family or relatives	249 (66.9)	
Health condition	Good	175 (47.1)	
	Moderate	145 (39.0)	
	Bad	52 (13.9)	
Socioeconomic status	High	85 (22.9)	
	Middle	162 (43.5)	
	Low	125 (33.6)	
Solo dining experience			3.17 (0.81)
Weekly solo dining frequency	Breakfast		2.77 (1.89)
	Lunch		3.70 (2.05)
	Dinner		2.84 (2.12)
Common place of solo dining	Restaurant	82 (22.0)	
	Convenience store	16 (4.3)	
	Home	254 (68.3)	
	Lecture room/school lounge	14 (3.8)	
	Other	6 (1.6)	
Major reason for solo dining ^1^	To save time	185 (17.7)	
	Comfort	247 (23.7)	
	No one to eat with	208 (19.9)	
	Save money	171 (16.4)	
	Weight loss/restricted diet	56 (5.4)	
	Do not feel like meeting people	122 (11.7)	
	Social distancing owing to COVID-19	55 (5.3)	
Perception of solo dining	Positive	171 (46.0)	
	Neither	196 (52.7)	
	Negative	5 (1.3)	
Satisfaction with solo dining	Satisfied	283 (76.1)	
	Neither	80 (21.5)	
	Not satisfied	9 (2.4)	

M: mean, SD: standard deviation, COVID-19: coronavirus disease 2019. ^1^ Participants could choose up to three reasons, and 1044 responses were collected.

**Table 2:** Association between solo dining-related characteristics and the occurrence of depression according to participants’ general characteristics (*N = 372*).

Variable	Category	Solo Dining Frequency M (SD)	*t*/*F*(*p*)	Perception of Solo Dining *n* (%)			χ^2^ (*p*)	Satisfaction with Solo Dining M (SD)	*t*/*F*(*p*)	Depression	*t*/*F*(*p*)
				Positive	Neither	Negative					
Gender	Male	8.70 (4.38)	0.55 (0.583)	84 (45.2)	99 (53.2)	3 (1.6)	0.27 (0.872)	3.89 (0.68)	0.75 (0.456)	15.76 (10.55)	3.14 (0.002) **
	Female	8.45 (4.49)		87 (46.8)	97 (52.2)	2 (1.1)		3.94 (0.71)		19.13 (10.14)	
Year	1 (a)	7.52 (4.75)	1.49 (0.218)	28 (45.9)	33 (54.1)	0 (0)	6.77 (0.342)	3.80 (0.70)	1.51 (0.212)	16.62 (11.32)	4.59(0.004) ** ^1^ b < c (0.023), b < d (0.013)
	2 (b)	8.76 (4.18)		43 (49.4)	44 (50.6)	0 (0)		4.03 (0.69)		14.18 (9.25)	
	3 (c)	8.57 (4.65)		47 (51.6)	42 (46.2)	2 (2.2)		3.92 (0.69)		19.00 (10.37)	
	4 (d)	8.93 (4.27)		53 (39.8)	77 (57.9)	3 (2.3)		3.88 (0.70)		18.89 (10.37)	
School registration status	Registered	8.68 (4.58)	1.03 (0.304)	145 (46.5)	162 (51.9)	5 (1.6)	1.28 (0.536)	3.91 (0.68)	0.24 (0.814)	17.34 (10.33)	0.47 (0.638)
	On leave	8.03 (3.56)		26 (43.3)	34 (56.7)	0 (0)		3.93 (0.76)		18.03 (11.25)	
Living arrangement	Live in a dormitory (a)	8.78 (4.33)	12.96 (<0.001) *** ^1^ b > a (0.049), b > c (0.001), b > d (<0.001)	20 (54.1)	17 (45.9)	0 (0)	10.64 (0.100)	3.89 (0.77)	0.66 (0.576)	18.16 (10.60)	1.43 (0.233)
	Live alone (b)	11.19 (4.12)		37 (51.4)	33 (45.8)	2 (2.8)		3.99 (0.76)		15.72 (10.18)	
	Live with friends (c)	5.93 (4.20)		3 (21.4)	10 (71.4)	1 (7.1)		3.71 (0.73)		14.14 (10.85)	
	Live with family or relatives (d)	7.93 (4.25)		111 (44.6)	136 (54.6)	2 (0.8)		3.91 (0.66)		18.03 (10.49)	
Health condition	Good (a)	8.20 (4.46)	3.86 (0.022) * ^1^ c > a (0.023)	85 (48.6)	88 (50.3)	2 (1.1)	1.31 (0.860)	3.96 (0.70)	0.73 (0.484)	14.25 (9.33)	19.17 (<0.001) *** ^1^ a < b (<0.001), a < c (<0.001)
	Moderate (b)	8.47 (4.47)		65 (44.8)	78 (53.8)	2 (1.4)		3.88 (0.69)		19.40 (10.51)	
	Bad (c)	10.12 (3.96)		21 (40.4)	30 (57.7)	1 (1.9)		3.87 (0.72)		22.79 (10.67)	
Socioeconomic status	High	8.41 (4.26)	1.82 (0.163)	43 (50.6)	40 (47.1)	2 (2.4)	5.30 (0.258)	3.87 (0.74)	0.33 (0.717)	17.32 (10.77)	1.75 (0.175)
	Middle	8.19 (4.53)		75 (46.3)	87 (53.7)	0 (0)		3.94 (0.66)		16.48 (10.27)	
	Low	9.18 (4.39)		53 (42.4)	69 (55.2)	3 (2.4)		3.90 (0.71)		18.80 (10.47)	

M: mean, SD: standard deviation. ^1^ Scheffe’s post hoc test, * *p* < 0.05, ** *p* < 0.01, *** *p* < 0.001.

**Table 3:** Correlations among solo dining-related characteristics and self- and non-self-determined solitude and depression (*N* = 372).

		Age	Solo Dining Experiences	Solo Dining Frequency (Weekly)				Satisfaction with Solo Dining	SDS	NSDS	Depression
				Breakfast	Lunch	Dinner	Total				
		*r* (*p*)	*r* (*p*)	*r* (*p*)	*r* (*p*)	*r* (*p*)	*r* (*p*)	*r* (*p*)	*r* (*p*)	*r* (*p*)	*r* (*p*)
Age		1									
Solo dining experiences		0.278 (<0.001) ***	1								
Solo dining frequency (weekly)	Breakfast	0.032 (0.601)	0.133 (0.028) *	1							
	Lunch	0.110 (0.034) *	0.089 (0.086)	0.256 (<0.001) ***	1						
	Dinner	0.177 (0.001) **	0.143 (0.006) **	0.204 (0.001) **	0.507 (<0.001) ***	1					
	Total	0.112 (0.030) *	0.154 (0.003) **	0.636 (<0.001) ***	0.776 (<0.001) ***	0.773 (<0.001) ***	1				
Satisfaction with solo dining		−0.018 (0.725)	0.098 (0.058)	−0.010 (0.868)	0.052 (0.316)	0.103 (0.048) *	0.090 (0.080)	1			
SDS		−0.114 (0.028) *	0.171 (0.001) **	0.052 (0.388)	0.023 (0.656)	0.091 (0.079)	0.098 (0.059)	0.460 (<0.001) ***	1		
NSDS		−0.014 (0.783)	−0.030 (0.559)	0.147 (0.015) *	0.044 (0.398)	0.013 (0.808)	0.074 (0.154)	−0.098 (0.060)	0.065 (0.214)	1	
Depression		0.098 (0.059)	0.008 (0.882)	0.143 (0.019) *	0.156 (0.002) **	0.104 (0.045) *	0.139(0.007) **	−0.209 (<0.001) ***	−0.134 (0.009) **	0.563 (<0.001) ***	1

SDS: self-determined solitude, NSDS: non-self-determined solitude, * *p* < 0.05, ** *p* < 0.01, *** *p* < 0.001.

**Table 4:** Factors influencing depression (*N* = 372).

Independent Variable		B	SE	Β	*T*	*p*	VIF	95% CI	
								Lower Limit	Upper Limit
Constant		9.45	4.12		2.30	0.022 *		1.36	17.54
Gender ^1^		−2.47	0.87	−0.12	2.83	0.005 **	1.08	−4.19	−0.76
Year		0.67	0.39	0.07	1.70	0.090	1.05	−0.10	1.44
Health condition ^2^		2.16	0.63	0.15	3.44	0.001 **	1.12	0.93	3.40
Solo dining	Breakfast	−0.02	0.21	−0.00	0.07	0.943	1.07	−0.44	0.41
	Lunch	0.50	0.24	0.10	2.06	0.040 *	1.40	0.02	0.98
	Dinner	0.19	0.24	0.04	0.80	0.424	1.41	−0.28	0.65
Satisfaction with solo dining		−1.44	0.69	−0.10	2.08	0.038 *	1.31	−2.80	−0.08
Self-determined solitude		−0.13	0.04	−0.15	3.17	0.002 **	1.36	−0.22	−0.05
Non-self-determined solitude		0.38	0.03	0.51	12.00	<0.001 ***	1.11	0.32	0.44
Adjusted R^2^ = 0.403									
F = 28.85, *p* < 0.001									

Durbin–Watson coefficient = 2.085, Durbin–Watson’s du (lower critical limit) = 1.880, 4-du (upper critical limit) = 2.120. ^1^ Female; ^2^ good condition. SE: standard error, VIF: variance inflation factor, CI: confidence interval. * *p* < 0.05, ** *p* < 0.01, *** *p* < 0.001.

## Data Availability

The data presented in this study are available on request from the corresponding author and with permission of the Institutional Review Board of Chung-Ang University.
